# The Dietary Flavonoid Kaempferol Mediates Anti-Inflammatory Responses via the Src, Syk, IRAK1, and IRAK4 Molecular Targets

**DOI:** 10.1155/2015/904142

**Published:** 2015-04-02

**Authors:** Shi Hyoung Kim, Jae Gwang Park, Jongsung Lee, Woo Seok Yang, Gye Won Park, Han Gyung Kim, Young-Su Yi, Kwang-Soo Baek, Nak Yoon Sung, Muhammad Jahangir Hossen, Mi-nam Lee, Jong-Hoon Kim, Jae Youl Cho

**Affiliations:** ^1^Department of Genetic Engineering, Sungkyunkwan University, Suwon 440-746, Republic of Korea; ^2^Department of Dermatological Health Management, Eulji University, Seongnam, Republic of Korea; ^3^Department of Food Science and Biotechnology, Sungkyunkwan University, Suwon 440-746, Republic of Korea; ^4^Department of Animal Science, Patuakhali Science and Technology University, Patuakhali 8602, Bangladesh; ^5^Department of Food and Nutrition, School of Food Service Industry, Chungkang College of Cultural Industries, Icheon 467-744, Republic of Korea; ^6^Department of Veterinary Physiology, College of Veterinary Medicine, Biosafety Research Institute, Chonbuk National University, Jeonju 561-756, Republic of Korea

## Abstract

Even though a lot of reports have suggested the anti-inflammatory activity of kaempferol (KF) in macrophages, little is known about its exact anti-inflammatory mode of action and its immunopharmacological target molecules. In this study, we explored anti-inflammatory activity of KF in LPS-treated macrophages. In particular, molecular targets for KF action were identified by using biochemical and molecular biological analyses. KF suppressed the release of nitric oxide (NO) and prostaglandin E_2_ (PGE_2_), downregulated the cellular adhesion of U937 cells to fibronectin (FN), neutralized the generation of radicals, and diminished mRNA expression levels of inflammatory genes encoding inducible NO synthase (iNOS), TNF-*α*, and cyclooxygenase- (COX-) 2 in lipopolysaccharide- (LPS-) and sodium nitroprusside- (SNP-) treated RAW264.7 cells and peritoneal macrophages. KF reduced NF-*κ*B (p65 and p50) and AP-1 (c-Jun and c-Fos) levels in the nucleus and their transcriptional activity. Interestingly, it was found that Src, Syk, IRAK1, and IRAK4 responsible for NF-*κ*B and AP-1 activation were identified as the direct molecular targets of KF by kinase enzyme assays and by measuring their phosphorylation patterns. KF was revealed to have *in vitro* and *in vivo* anti-inflammatory activity by the direct suppression of Src, Syk, IRAK1, and IRAK4, involved in the activation of NF-*κ*B and AP-1.

## 1. Introduction

Inflammation is an innate immune response that protects the human body from chemicals and infectious microorganisms [1]. This response is comprised of pain, heat, swelling, and redness. At the molecular level, various cytokines (e.g., tumor necrosis factor- (TNF-) *α*), hydrolytic enzymes, toxic molecules (e.g., nitric oxide (NO) and reactive oxygen species (ROS)), and mediators (e.g., prostaglandin E_2_ (PGE_2_)) are released from inflammatory cells [2, 3]. Many complicated biochemical processes are required to trigger the cellular inflammatory response. The activation of pattern recognition receptors (e.g., Toll-like receptors (TLRs)) is reliant on association with counter ligands such as lipopolysaccharide (LPS) and peptidoglycan (PGN) [4]. Then, many different intracellular signaling cascades are initiated via two major adaptor molecules (Toll/Il-1 receptor-domain-containing, adapter-inducing interferon-*β* (TRIF), and myeloid differentiation response gene 88 (MyD88)) generated to eventually activate inflammation-regulatory transcription factors including nuclear factor- (NF-) *κ*B and activator protein- (AP-) 1 to express inflammation-mediating genes encoding inducible NO synthase (iNOS), cyclooxygenase-2 (COX-2), cytokines, and chemokines [5, 6].

Even though inflammatory events are one of critical defensive ways in human body, prolonged levels of inflammation somehow cause organ damage leading to loss of functions and their related diseases such as cancer, diabetes, and atherosclerosis [7, 8]. However, the knowledge as to how inflammation can induce tissue damage is still not fully understood. Oxidative stress accompanied by sustained inflammation is considered as major causing factor in generation of organ damage. This led us to the fact that antioxidative agents could be functional in preventing such damage [9]. Since toxic radicals are not the only factor inducing inflammation-mediated functional damage, it is needed that other pathological pathways such as cellular inflammatory signaling should be also targeted for treating chronic inflammatory diseases [10, 11]. These points indicate that anti-inflammatory remedy to treat acute and chronic inflammatory responses should include additional pharmacological action to radical scavenging activity.

**Figure 1:**
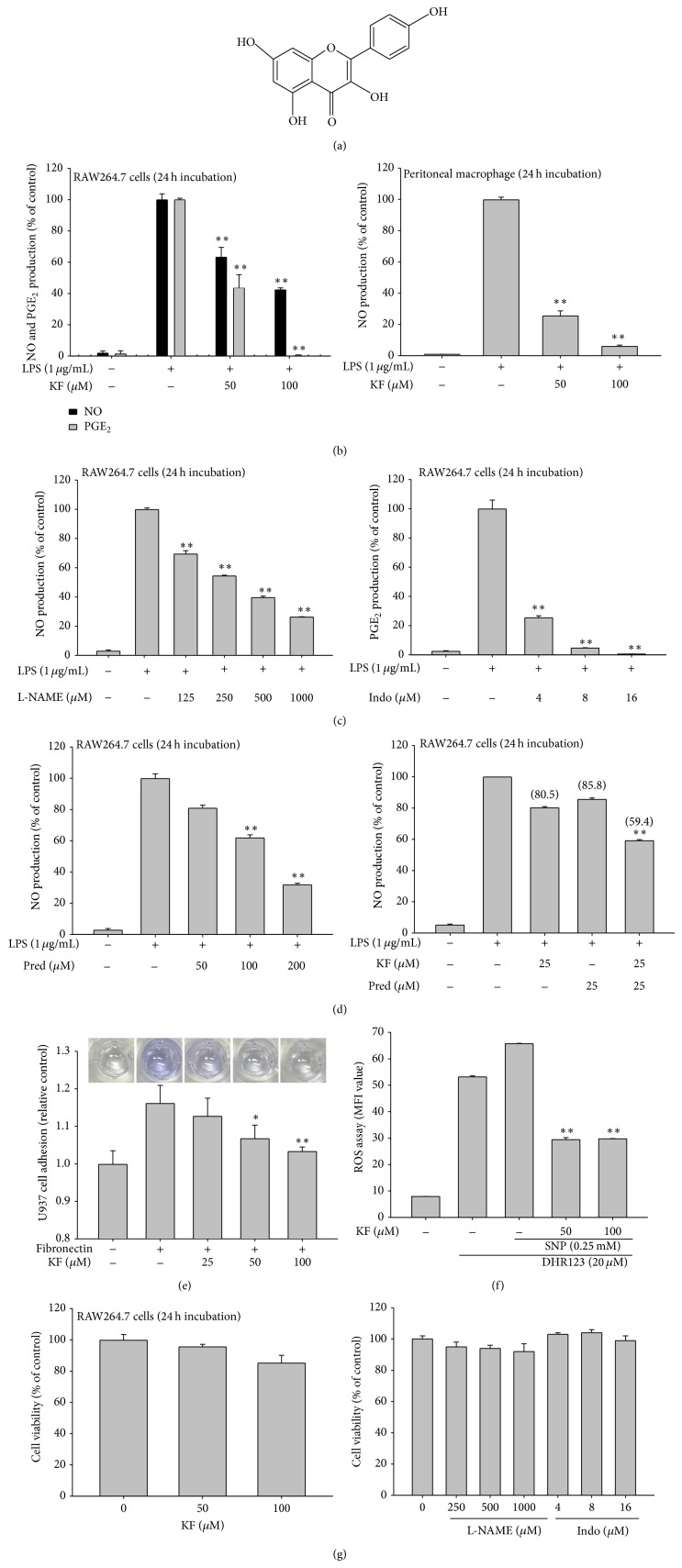
The effects of KF on the production of NO, PGE_2_, and TNF-*α* in macrophages, the neutralization of ROS, and macrophage viability. (a) Chemical structure of KF. (b, c, and d) RAW264.7 cells or peritoneal macrophages (1 × 10^6^ cells/mL) were incubated with LPS (1 *μ*g/mL) under either single treatment (b, c, and d left panel) of KF, Indo, L-NAME, and Pred or combination treatment (d right panel) of KF with Pred for 24 h. The supernatants were collected and the NO or PGE_2_ concentrations in the supernatants were determined using the Griess assay or EIA. (e) Effect of KF on cell-matrix protein adhesion was evaluated by using fibronectin (FN) coated conditions. U937 cells were pretreated with KF and seeded on FN (50 *μ*g/mL) coated plates for 4 h. The number of attached cells was determined by crystal violet staining. (f) RAW264.7 cells preincubated with KF were treated with DHR123 (20 *μ*M) in the presence or absence of SNP (0.25 mM) for 2 h. The level of radicals was determined by flow cytometric analysis. (g) RAW264.7 cells (1 × 10^6^ cells/mL) were treated with KF or standard compounds (Indo and L-NAME) for 24 h. Cell viability was evaluated using the MTT assay. All of the data are expressed as the mean ± SD of experiments that were performed with six samples. ^∗^ *P* < 0.05 and ^∗∗^ *P* < 0.01 compared to the normal or control groups.

Kaempferol (3,5,7-trihydroxy-2-(4-hydroxyphenyl)-4H-1-benzopyran-4-one, KF, Figure 1(a)) is a representative polyphenolic compound in nature [12]. This compound is highly contained in most edible herbal plants such as tea, fruits, and vegetables [13]. These include* Allium cepa* (onion),* Camellia sinensis* (tea),* Citrus paradisi* (grapefruit),* Fragaria vesca* (strawberry),* Lactuca sativa* (lettuce), and* Morinda citrifolia* (Indian mulberry) as well as medicinal plants such as* Cerbera manghas* [13–15]. Owing to numerous pharmacological studies due to its popularity, it has been reported that this compound is able to display antioxidative, anticancer, anti-inflammatory, and antiaging properties [16–18]. By molecular approaches of KF, some of KF target proteins have been identified. For example, kaempferol was revealed to be skin protective by suppressing kinase activities of ribosomal S6 kinase, mitogen, and stress-activated protein kinase, which are activated by UV irradiation, via competition with ATP at ATP-binding pocket [19]. It was also proposed that kaempferol is effective in inhibiting cancer progression through antagonizing selective estrogen-related receptors alpha and gamma [20]. Furthermore, epidermal growth factor-induced neoplastic transformation of mouse epidermal JB6 P+ cells was reduced by KF through lowering phosphatidylinositol 3-kinase (PI3K) activity [21]. In contrast to these results, molecular targets identified in anti-inflammatory action of KF were not reported yet, although NF-*κ*B and AP-1 are known as target pathways of KF [22–26]. Thus, most of reports simply suggested that the phosphorylation of mitogen activated protein kinases (MAPK) such as extracellular-signal-regulated kinase 1/2 (ERK1/2), p38, and c-Jun N-terminal kinase (JNK) and the phosphorylation of inhibitor of *κ*B kinase (IKK*α*/*β*), which were critical steps for NF-*κ*B and AP-1 activations, were remarkably reduced by KF treatment in cellular and tissue levels [22]. In view of the fact that flavonoids can be considered as anti-inflammatory remedy due to their multiple pharmacological actions, it is important for us to understand as to which molecular targets can be contributed to their anti-inflammatory responses. In the present study, therefore, we aimed to expand the understanding levels of KF-mediated anti-inflammatory process by identifying the molecular targets regulating LPS-stimulated macrophages.

## 2. Materials and Methods

### 2.1. Materials

KF, indomethacin (Indo), prednisolone (Pred), N-nitro-L-arginine-methyl ester (L-NAME), ranitidine, polyethylenimine (PEI), arachidonic acid, 3-(4,5-dimethylthiazol-2-yl)-2,5-diphenyltetrazolium bromide (a tetrazole) (MTT), sodium nitroprusside (SNP), sodium dodecyl sulfate (SDS), dimethyl sulfoxide (DMSO), pam3CSK, dihydrorhodamine (DHR) 123, and lipopolysaccharide (LPS,* E. coli* 0111:B4) were purchased from Sigma Chemical Co. (St. Louis, MO). Piceatannol (Picea), PP2, SB203580 (SB), and SP600125 (SP) were obtained from Calbiochem (La Jolla, CA). The enzyme immune assay (EIA) kits that were used to determine PGE_2_ levels were purchased from Amersham (Little Chalfont, Buckinghamshire, UK). Fibronectin (FN), fetal bovine serum (FBS), penicillin, streptomycin, TRIzol Reagent, and RPMI1640 were obtained from GIBCO (Grand Island, NY). RAW264.7, U937, and HEK293 cells were purchased from ATCC (Rockville, MD). All other chemicals used in this study were of analytical grade of Sigma Chemical Co. Phosphospecific or total antibodies that were raised against p65, p50, c-Fos, c-Jun, inhibitor of *κ*B*α* (I*κ*B*α*), Src, spleen tyrosine kinase (Syk), p85, ERK, JNK, p38, mitogen activated protein kinase (MKK)3, MKK4, interleukin-1 receptor-associated kinase 1 (IRAK1), IRAK4, transforming growth factor *β*-activated kinase-1 (TAK1), Akt, I*κ*B*α*, Myc, lamin A/C, and *β*-actin were obtained from Cell Signaling (Beverly, MA).

### 2.2. Construction of Expression Vectors

All constructs were prepared by amplification using a typical culture method with competent* E. coli* (DH5*α*). FLAG-MyD88, CFP-TRIF, and Myc-Syk constructs were used as reported previously [27]. Luciferase constructs containing binding sites for NF-*κ*B and AP-1 were used as reported previously [28]. All constructs were confirmed by automated DNA sequencing.

### 2.3. Mice

Six-week-old C57BL/6 mice were purchased from DAEHAN BIOLINK (Chungbuk, Republic of Korea) and were housed in groups of 6–8 mice under a 12 h light/dark cycle (lights on at 6 am). Water and pellet diets (Samyang, Daejeon, Republic of Korea) were supplied* ad libitum*. Animals were cared for in accordance with the guidelines issued by the National Institute of Health for the Care and Use of Laboratory Animals (NIH Publication 80-23, revised in 1996). Studies were performed in accordance with guidelines established by the Institutional Animal Care and Use Committee at Sungkyunkwan University (Suwon, Republic of Korea; Approval ID: SKKUBBI 13-6-4).

### 2.4. Preparation of Peritoneal Macrophages

Peritoneal exudates were obtained from C57BL/6 male mice (7-8 weeks old and weighing 17–21 g) by lavage 4 days after intraperitoneal injection of 1 mL of sterile 4% thioglycollate broth (Difco Laboratories, Detroit, MI) as reported previously [29]. After the exudates were washed with RPMI1640 medium containing 2% FBS, peritoneal macrophages (1 × 10^6^ cells/mL) were plated in 100 mm tissue culture dishes for 4 h at 37°C in a 5% CO_2_ humidified atmosphere.

### 2.5. Cell Culture and Drug Preparation

RAW264.7 cells, a murine macrophage cell line, and HEK293 cells were maintained in RPMI1640 media supplemented with 100 U/mL of penicillin, 100 *μ*g/mL of streptomycin, and 10% FBS. The cells were grown at 37°C and 5% CO_2_ in humidified air. The stock solutions of KF for the* in vitro* experiments were prepared using DMSO.

### 2.6. Determination of NO and PGE_2_ Production

After preincubation of RAW264.7 cells or peritoneal macrophages (1 × 10^6^ cells/mL) for 18 h, the cells were treated with KF (0 to 100 *μ*M) or standard compounds (Pred, L-NAME or Indo) for 30 min and then further incubated with LPS (1 *μ*g/mL) for 24 h. The inhibitory effects of KF on NO and PGE_2_ production were determined by analyzing NO and PGE_2_ levels using Griess reagents and an EIA kit, as previously described [30, 31].

### 2.7. Cell Adhesion Assay

A U937 cell-fibronectin (FN) adhesion assay was performed as reported previously [32, 33]. U937 cells (5 × 10^5^ cells/well) pretreated with KF were seeded on a fibronectin (50 *μ*g/mL) coated plate and incubated for 4 h [34]. After removing the unbound cells with PBS, the attached cells were treated with 0.1% of crystal violet for 15 min. The OD at 570 nm was measured with a SpectraMax 250 microplate reader.

### 2.8. Determination of ROS Generation

The level of intracellular ROS was determined by a change in fluorescence resulting from the oxidation of the fluorescent probe, DHR123. Briefly, 5 × 10^5^ RAW264.7 cells were exposed to KF for 30 min. After incubation, cells were then incubated with SNP (0.25 mM), an inducer of ROS production, at 37°C for 2 h. Cells were incubated with 20 *μ*M of the fluorescent probe DHR123 for 1 h at 37°C. The degree of fluorescence, corresponding to intracellular ROS, was determined using a FACScan flow cytometer (Becton-Dickinson, San Jose, CA), as reported previously [3, 33, 35]. Briefly, the RAW264.7 cells treated with KF, SNP, and DHR123 were washed with a staining buffer (containing 2% rabbit serum and 1% sodium azide in PBS) and incubated for a further 45 min on ice. After washing three times with staining buffer, stained cells were analyzed on a FACScan flow cytometer (Becton-Dickinson, San Jose, CA, USA).

### 2.9. Cell Viability Test

After preincubation of RAW264.7 cells (1 × 10^6^ cells/mL) for 18 h, KF (0 to 100 *μ*M) or standard compounds (L-NAME or Indo) were added to the cell suspensions and incubated for 24 h. The cytotoxic effects of KF were then evaluated using a conventional MTT assay, as previously reported [36–38]. Three hours prior to culture termination, 10 *μ*L of MTT solution (10 mg/mL in phosphate-buffered saline, pH 7.4) was added, and the cells were continuously cultured until the termination of the experiment. The incubation was halted by the addition of 15% SDS into each well, solubilizing the formazan. The absorbance at 570 nm (OD_570–630_) was measured using a SpectraMax 250 microplate reader (BioTex, Bad Friedrichshall, Germany).

### 2.10. mRNA Analysis Using Quantitative Polymerase Chain Reactions

In order to determine cytokine mRNA expression levels, total RNA was isolated from LPS-treated RAW264.7 cells using TRIzol Reagent, according to the manufacturer's recommended instructions. Total RNA was stored at −70°C until use. Semiquantitative RT reactions were conducted as previously reported [39, 40]. Quantification of mRNA was performed by real-time RT-PCR with SYBR Premix Ex Taq according to the manufacturer's instructions (Takara, Shiga, Japan) using a real-time thermal cycler (Bio-Rad, Hercules, CA), as reported previously [41]. All of the primers (Bioneer, Daejeon, Republic of Korea) used are indicated in Table 1.

### 2.11. Preparation of Cell Lysates and Nuclear Fractions for Immunoblotting

RAW264.7 cells (5 × 10^6^ cells/mL) were washed 3 times in cold PBS containing 1 mM sodium orthovanadate and lysed in lysis buffer (20 mM Tris-HCl, pH 7.4, 2 mM EDTA, 2 mM ethyleneglycotetraacetic acid, 50 mM *β*-glycerophosphate, 1 mM sodium orthovanadate, 1 mM dithiothreitol, 1% Triton X-100, 10% glycerol, 10 *μ*g/mL aprotinin, 10 *μ*g/mL pepstatin, 1 mM benzimide, and 2 mM PMSF) for 30 min, with rotation, at 4°C. The lysates were clarified by centrifugation at 16,000 ×g for 10 min at 4°C and stored at −20°C until needed.

Nuclear lysates were prepared using a three-step procedure [42]. After treatment, the cells were collected with a rubber policeman, washed with 1 × PBS, and lysed in 500 *μ*L of lysis buffer containing 50 mM KCl, 0.5% Nonidet P-40, 25 mM HEPES (pH 7.8), 1 mM phenylmethylsulfonyl fluoride, 10 *μ*g/mL leupeptin, 20 *μ*g/mL aprotinin, and 100 *μ*M 1,4-dithiothreitol (DTT) on ice for 4 min. Cell lysates were then centrifuged at 14,000 rpm for 1 min in a microcentrifuge. During the second step, the pellet (the nuclear fraction) was washed once with wash buffer without Nonidet P-40. During the final step, the nuclei were treated with an extraction buffer containing 500 mM KCl, 10% glycerol, and several other reagents that were contained in the lysis buffer. The nuclei/extraction buffer mixture was frozen at −80°C and then thawed on ice and centrifuged at 14,000 rpm for 5 min. The supernatant was collected as the nuclear extract.

Whole cell or nuclear lysates were then analyzed using immunoblotting. Proteins were separated on 10% SDS-polyacrylamide gels and transferred by electroblotting to a polyvinylidene difluoride (PVDF) membrane. Membranes were blocked for 60 min in Tris-buffered saline containing 3% FBS, 20 mM NaF, 2 mM EDTA, and 0.2% Tween 20 at room temperature. The membranes were incubated for 60 min with specific primary antibodies at 4°C, washed 3 times with the same buffer, and incubated for an additional 60 min with HRP-conjugated secondary antibodies. The total and phosphorylated levels of p65, p50, c-Fos, c-Jun, I*κ*B*α*, Src, Syk, p85, ERK, JNK, p38, MKK3, MKK4, IRAK1, IRAK4, TAK1, Akt, I*κ*B*α*, Myc, lamin A/C, and *β*-actin were visualized using an ECL system (Amersham, Little Chalfont, Buckinghamshire, UK), as reported previously [43].

### 2.12. DNA Transfection and Luciferase Reporter Gene Activity Assay

Overexpression experiment was performed with HEK293 cells (1 × 10^6^ cells/mL) by transfection of Myc-Syk (1 *μ*g/mL) using the PEI method in 12-well plates, as reported previously [44, 45]. The cells were utilized for the experiments 24 h after transfection. KF was additionally treated to the cells before 12 h of termination. For reporter gene assay, HEK293 cells (1 × 10^6^ cells/mL) were transfected with 1 *μ*g of plasmids containing NF-*κ*B-Luc, or AP-1-Luc, as well as *β*-galactosidase, using the PEI method in 12-well plates, according to the procedure that was outlined in a previous report [44, 45]. Luciferase assays were performed using the Luciferase Assay System (Promega, Madison, WI), as previously reported [46].

### 2.13. *In Vitro* Kinase Assay with Purified Enzymes

In order to evaluate the inhibition of the kinase activities of Src, Syk, IRAK1, or IRAK4 using purified enzymes, the kinase profiler service from Millipore (Billerica, MA) was used. Purified Src, Syk, IRAK1, or IRAK4 (human) (1–5 mU) were incubated with the reaction buffer in a final reaction volume of 25 *μ*L. The reaction was initiated by the addition of MgATP. After incubation for 40 min at room temperature, the reaction was stopped by the addition of 5 mL of a 3% phosphoric acid solution. Ten microliters of the reaction was then spotted onto a P30 Filtermat and washed three times for 5 min in 75 mM phosphoric acid and once in methanol prior to drying and scintillation counting.

### 2.14. Statistical Analyses

All of the data presented in this paper are expressed as the means ± SD of experiments. For the statistical comparisons, the results were analyzed using either ANOVA/Scheffe's* post hoc* test or the Kruskal-Wallis/Mann-Whitney test. A *P* value <0.05 was considered to be a statistically significant difference. All of the statistical tests were carried out using the computer program SPSS (SPSS Inc., Chicago, IL). Similar experimental data were also observed using an additional independent set of* in vitro* experiments that was conducted using the same numbers of samples or mice.

## 3. Results

### 3.1. Effect of KF on the Inflammatory Response

KF (50 and 100 *μ*M) inhibited the production of NO (26.7 *μ*M as nitrite) and PGE_2_ (3.4 ng/mL) in LPS-treated RAW264.7 cells relative to basal levels of NO (0.53 *μ*M) and PGE_2_ (0.098 ng/mL) in resting cells up to 98% in a dose-dependent manner (Figure 1(b) left panel). Similarly, increased levels of NO (55.2 *μ*M) in LPS-stimulated peritoneal macrophages relative to basal levels (0.39 *μ*M) were also clearly reduced by KF (Figure 1(b) right panel). Standard compounds (Pred, L-NAME, and Indo) also displayed a clear dose-dependent inhibitory pattern under the same NO and PGE_2_ production conditions (Figures 1(c) and 1(d) left panel), as reported previously [47], indicating that our experimental conditions were consistent with the literature. Interestingly, combination treatment (40.6% as percentage inhibition) of KF with Pred displayed additive inhibitory activity compared to single treatment of these compounds (KF (19.5%) and Pred (14.2%)) (Figure 1(d) right panel), indicating that the inhibitory mode of action by KF might be different from that of glucocorticoid drugs (e.g., prednisolone). Moreover, KF also dose-dependently downregulated the adhesion of U937 cells to FN (Figure 1(e)). Finally, since KF is a representative antioxidant flavonoid, we also confirmed its radical scavenging activity using SNP-induced ROS generation in RAW264.7 cells. Expectedly, KF showed strong antioxidative activity at both 50 and 100 *μ*M (Figure 1(f)), suggesting that these doses of KF are pharmacologically effective. Finally, MTT assays were used to determine if KF and other drugs suppress the production of NO, PGE_2_, and other radicals without altering cell cytotoxicity. As Figure 1(g) shows, there was no significant reduction in cell viability by the drugs, implying that the above effects were not derived by nonspecific cytotoxicity.

### 3.2. Effect of KF on Transcriptional Activation of the Inflammatory Response

**Figure 2:**
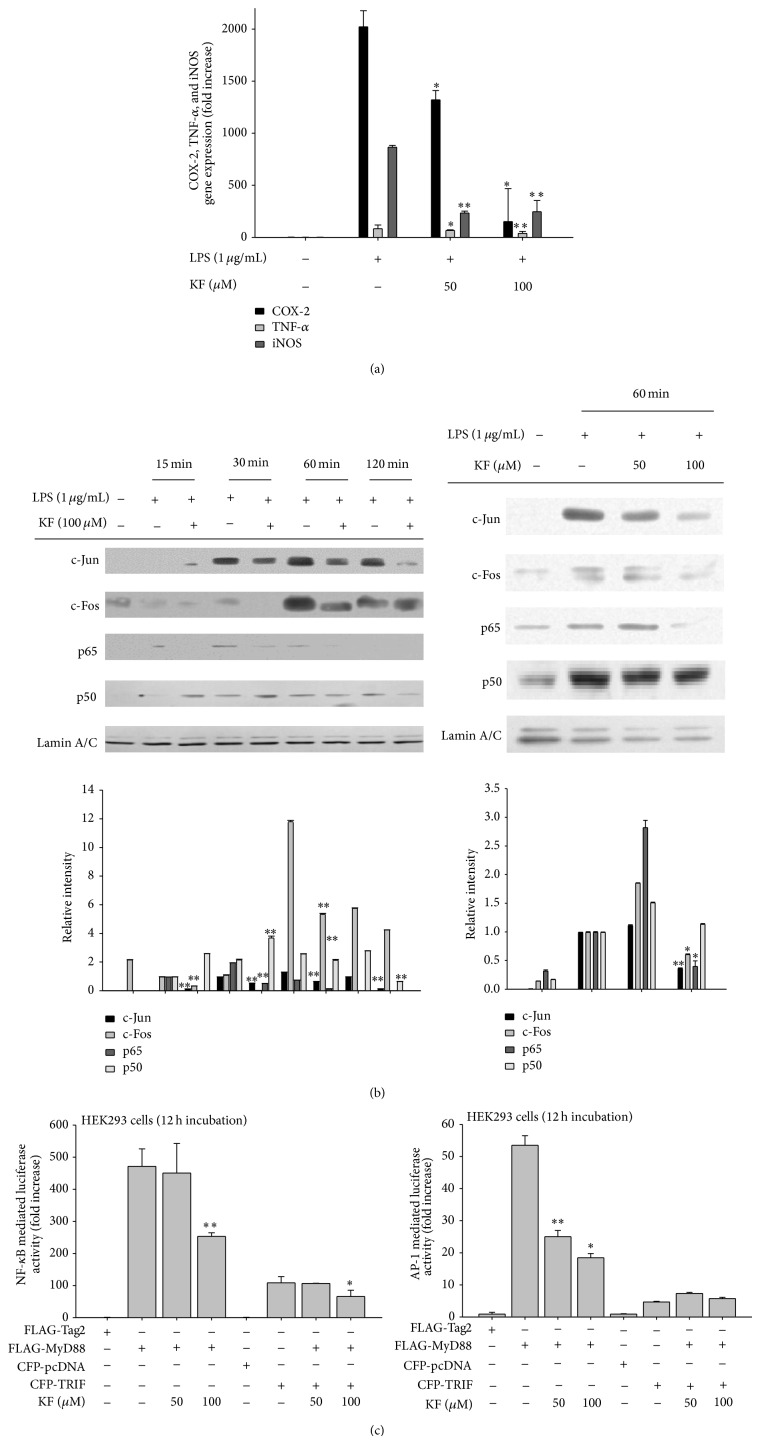
The effects of KF on iNOS, COX-2, and TNF-*α* gene expression and transcriptional regulation in LPS-treated RAW264.7 cells. (a) RAW264.7 cells (5 × 10^6^ cells/mL) were incubated with LPS (1 *μ*g/mL) in the presence or absence of KF for 6 h. iNOS, COX-2, and TNF-*α* mRNA levels were determined using real-time PCR. (b) RAW264.7 cells (5 × 10^6^ cells/mL) were incubated with LPS (1 *μ*g/mL) in the presence or absence of KF for the indicated times. After preparing the nuclear fractions, the translocated levels of total transcription factors (p65, p50, c-Fos, and c-Jun) were identified using immunoblot analyses. (c) HEK293 cells cotransfected with the NF-*κ*B-Luc or AP-1-Luc (1 *μ*g/mL each) and *β*-gal (as a transfection control) plasmid constructs were treated with KF in the presence or absence of adaptor molecule (MyD88 or TRIF) for 12 h. Luciferase activity was determined using luminometry. Relative intensity was calculated using total levels by the DNR Bio-Imaging System. All of the data are expressed as the mean ± SD of experiments that were performed with six or three (b) samples. ^∗^ *P* < 0.05 and ^∗∗^ *P* < 0.01 compared to the control group.

Since KF blocked the release of inflammatory mediators from LPS-stimulated macrophages, we next examined whether the inhibition occurred at the transcriptional level. For this purpose, the mRNA levels of inflammatory genes were measured by real-time PCR. As Figure 2(a) depicts, KF inhibited the expression of genes encoding COX-2, TNF-*α*, and iNOS in a dose-dependent manner. In agreement with this result, KF suppressed the nuclear levels of major transcription factors (c-Jun at 30, 60, and 120 min; c-Fos at 30 and 60 min; p65 at 15, 30, and 60 min; and p50 at 120 min) in LPS-treated RAW264.7 cells (Figure 2(b) left panel). At 60 min, the nuclear levels of c-Jun, c-Fos, and p65 were reduced by KF in a dose-dependent manner as well (Figure 2(b) right panel). Based on luciferase reporter gene assays, it was revealed that the transcriptional regulatory activity of NF-*κ*B and AP-1 was suppressed by 50 and 100 *μ*M of KF (Figure 2(c)), indicating that KF modulates the DNA binding ability of NF-*κ*B and AP-1.

### 3.3. Effect of KF on Upstream Signaling for NF-*κ*B and AP-1 Activation

**Figure 3:**
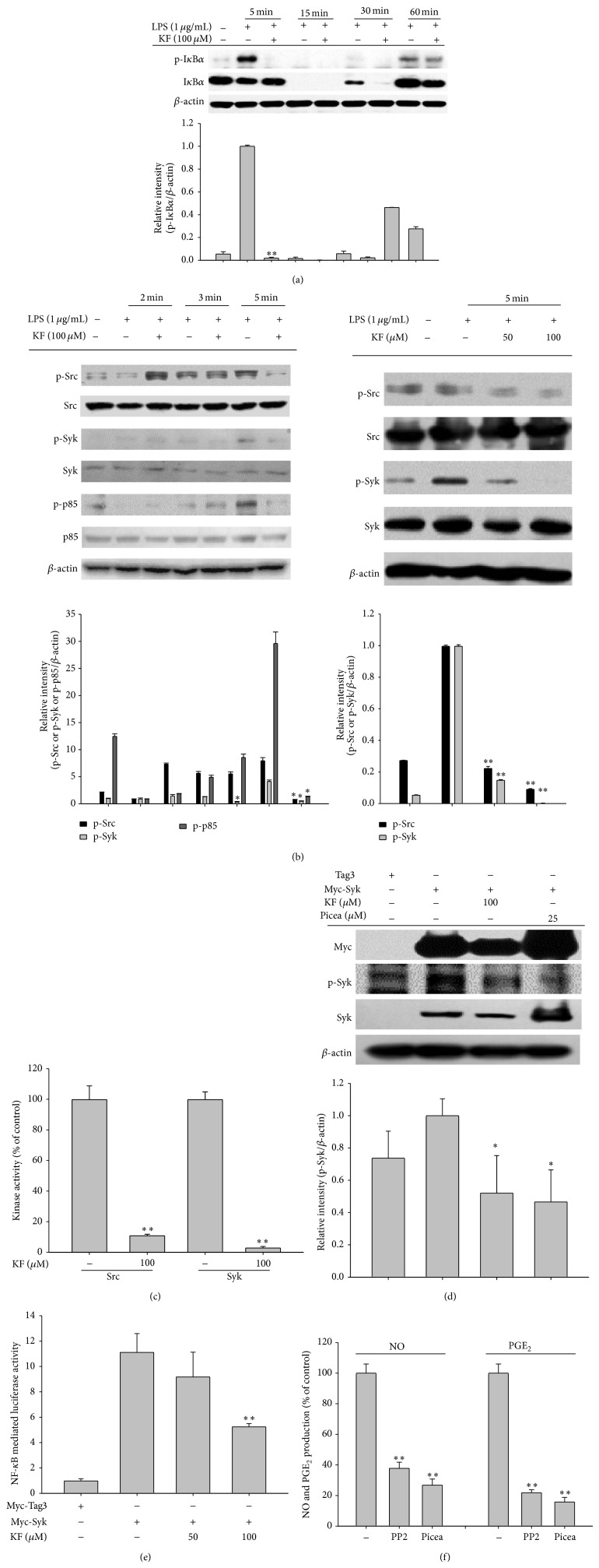
The effects of KF on NF-*κ*B activation signaling. (a and b) RAW264.7 cells (5 × 10^6^ cells/mL) were incubated with LPS (1 *μ*g/mL) in the presence or absence of KF for the indicated times. After preparing the whole lysates, the levels of total or phosphorylated I*κ*B*α*, Src, Syk, and p85 were identified using immunoblot analyses. (c) The inhibitory effects of KF on Src and Syk activity were determined using a conventional kinase assay with purified Src and Syk. (d) HEK293 cells transfected with Myc-Syk cDNA (1 *μ*g/mL) for 24 h were treated with KF or Picea for 12 h. After preparing the whole lysates, the levels of total or phosphorylated Myc, Syk, and *β*-actin were identified using immunoblot analyses. (e) HEK293 cells cotransfected with the NF-*κ*B-Luc (1 *μ*g/mL each) and *β*-gal (as a transfection control) plasmid constructs were treated with KF in the presence or absence of Myc-Syk for 12 h. Luciferase activity was determined using luminometry. (f) The inhibitory effects of PP2 or Picea on the production of NO or PGE_2_ were examined using the Griess assay and EIA. Relative intensity was calculated using total levels by the DNR Bio-Imaging System. All of the data are expressed as the mean ± SD of experiments that were performed with six or three (a, b, c, and d) samples. ^∗^ *P* < 0.05 and ^∗∗^ *P* < 0.01 compared to the control group.

After confirming that both the nuclear translocation of p65 and p50 and their promoter binding activities were strongly suppressed (Figure 2), we next examined the effect of KF on upstream signaling for NF-*κ*B activation. First, the time dependent inhibitory pattern of I*κ*B*α* phosphorylation was investigated [39, 48]. Interestingly, KF decreased the phosphorylation of I*κ*B*α* at 5 min and marginally suppressed this activity at 30 min (Figure 3(a)). Since the early phosphorylation of I*κ*B*α* is mediated by the early activation of protein tyrosine kinases Syk and Src, we confirmed the inhibitory activity of KF on the autophosphorylation patterns of Syk and Src. As Figure 3(b) shows, the phosphorylation of Src and Syk was suppressed by KF at 5 min. Moreover, the phosphorylation of p85/PI3K, a downstream substrate of Syk and Src involved in regulating the NF-*κ*B activation pathway [2, 49, 50], was also similarly diminished (Figure 3(b) left panel), indicating that the Syk/Src-mediated NF-*κ*B activation pathway could be targeted by KF. To determine whether KF is able to directly suppress the kinase activities of Src and Syk, enzyme assays were conducted with purified Src and Syk. Intriguingly, 100 *μ*M of KF clearly blocked the activity of these enzymes (Figure 3(c)). Using an overexpression strategy with Syk, which showed an increased kinase inhibitory pattern, we also validated that increased autophosphorylation levels of Syk from overexpressed Myc-Syk were also decreased by KF similar to inhibition levels by the Syk inhibitor piceatannol (Picea) (Figure 3(d)). In addition, Syk-induced NF-*κ*B mediated luciferase activity was also reduced by KF (Figure 3(e)), indicating that Syk can be directly suppressed by KF at the enzyme and related functional levels. Meanwhile, the PP2 and Picea, inhibitors of Src and Syk, respectively, exerted clear anti-inflammatory effects by diminishing the production levels of NO and PGE_2_ (Figure 3(f)).

**Figure 4:**
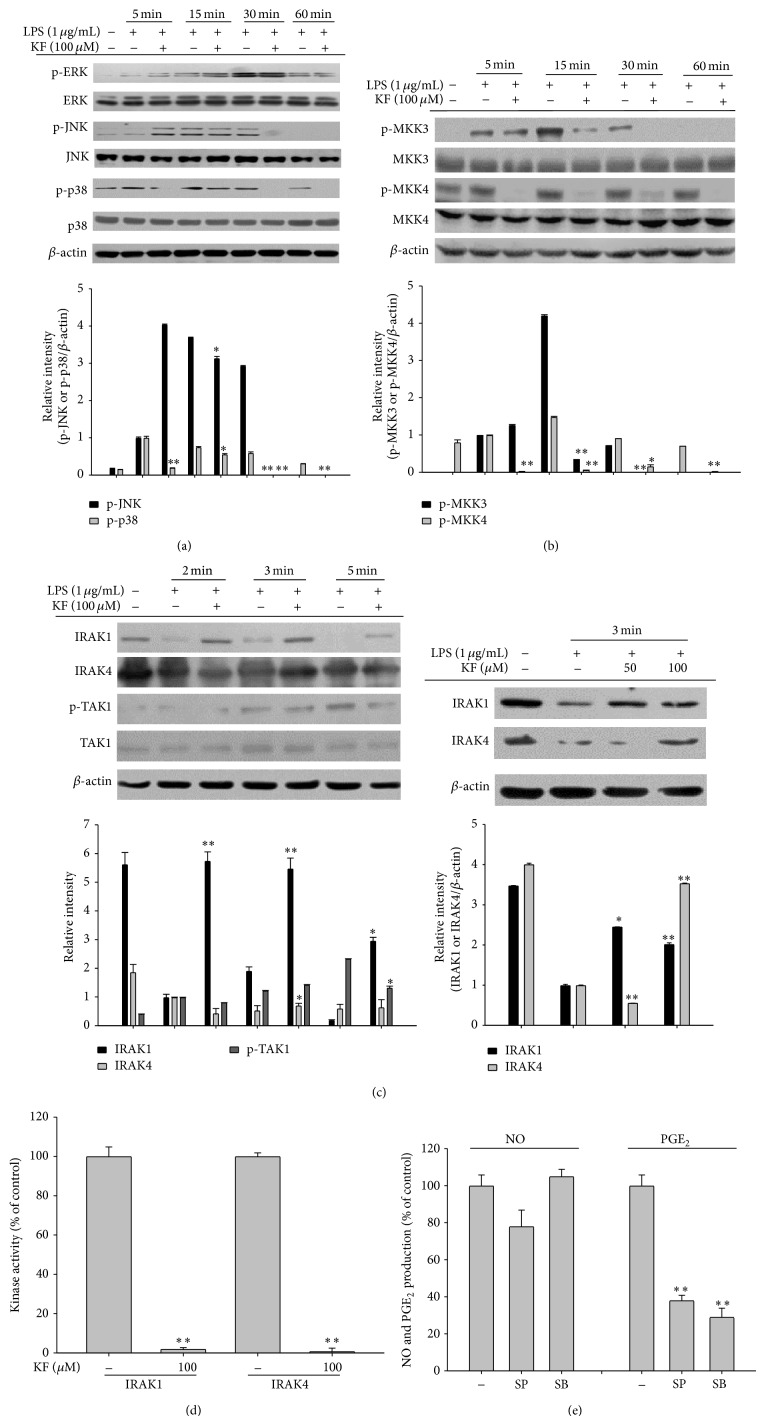
The effects of KF on AP-1 activation signaling. (a, b, and c) RAW264.7 cells (5 × 10^6^ cells/mL) were incubated with LPS (1 *μ*g/mL) in the presence or absence of KF for the indicated times. After preparing the whole lysates, the levels of total or phosphorylated ERK, JNK, p38, MKK3, MKK4, IRAK1, IRAK4, and TAK1 were identified using immunoblot analyses. (d) The inhibitory effects of KF on IRAK1 and IRAK4 activity were determined using a conventional kinase assay with purified IRAK1 and IRAK4. (e) The inhibitory effects of SB or SP on the production of NO or PGE_2_ were examined using the Griess assay and EIA. Relative intensity was calculated using total levels by the DNR Bio-Imaging System. All of the data are expressed as the mean ± SD of experiments that were performed with six or three (a, b, c, and d) samples. ^∗^ *P* < 0.05 and ^∗∗^ *P* < 0.01 compared to the control group.

Since LPS-induced translocation of c-Jun and c-Fos (Figure 2(b) left panel) and MyD88-dependent AP-1 activation were reduced by KF, the inhibitory effect of KF on upstream signaling for AP-1 activation was examined. As Figure 4(a) shows, the phosphorylation of JNK and p38 was inhibited by KF at 30 and 60 min, indicating that the activity of upstream kinases for JNK and p38 could also be regulated by KF. In fact, KF suppressed the phosphorylation of MKK3 and MKK4 kinases at 5, 15, 30, and 60 min (Figure 4(b)). Because IRAK1, IRAK4, and TAK1 are known as the upstream enzymes responsible for phosphorylating MAPK kinases [51], although these enzymes are also involved in the activation of NF-*κ*B pathway, we investigated the phosphorylation pattern of these proteins. Interestingly, the level of phospho-TAK1 was diminished at 5 min, while the degradation of IRAK1 and IRAK4 at 2 and 3 min by LPS was restored by treatment with 50 and 100 *μ*M of KF, respectively (Figure 4(c)). Finally, the ability of KF to directly suppress IRAK1 and IRAK4 enzyme activity was examined using enzyme assays. As expected, KF blocked the catalytic activity of IRAK1 and IRAK4 (Figure 4(d)), implying that these enzymes are directly targeted and the protein tyrosine kinases Src and Syk were suppressed (Figure 3(c)). Furthermore, inhibitors (SB203580 and SP600125) of p38 and JNK pathways significantly inhibited the production of PGE_2_ but not NO (Figure 4(e)), indicating that suppression of p38 and JNK by KF could contribute to the decrease in PGE_2_ production.

## 4. Discussion

KF, an abundant flavonoid, is involved in two target inhibitory pathways in inflammation-inducing macrophages. KF inhibited the nuclear translocation of the redox-specific transcription factors, NF-*κ*B and AP-1 (Figure 2(b)), which play critical roles in the induction of inflammatory genes [52]. These results strongly suggest that the upstream enzymes regulating the translocational activation of NF-*κ*B and AP-1 might be directly targeted by KF. In fact, immunoblotting analyses, molecular biological approaches, and kinase assays strongly indicate that KF is able to directly suppress the kinase activities of Src, Syk, IRAK1, and IRAK4 (Figures 3(c) and 4(d)). In addition, a suppressive activity of KF was linked to the suppression of subsequent downstream pathways comprised of I*κ*B*α* or MKK3/4, JNK, and p38 (Figures 3(a) and 4(a), top panel, and Figure 4(b)), which are involved in the modulation of NF-*κ*B and AP-1 activation [2, 49].

Thus far, only a few papers have reported the molecular pharmacological targets of KF. It was reported that the 90 kDa ribosomal S6 kinase (RSK) and mitogen and stress-activated protein kinase (MSK) proteins are directly suppressed by KF [19]. Silent information regulator 2 (SIR2), a member of the sirtuin family of NAD^+^-dependent histone deacetylases, was also found as a direct target protein of KF [53]. KF also suppressed the kinase activity of right open reading frame-2 protein kinase [54]. Phosphatidylinositol 3-kinase was also previously identified as a strong target of KF [21]. In addition to protein kinases, cdc25A tyrosine phosphatase was also inhibited by KF [55]. As a nonsignaling enzyme, fatty acid amide hydrolase was also revealed to be suppressed by KF [56]. Through a direct enzyme assay, we found additional enzymes, including Syk, Src, IRAK1, and IRAK4 (Figures 3(c) and 4(d)), which are involved in the inflammatory signaling events of activated macrophages and monocytes to regulate their production of inflammatory cytokines [2, 49] and *β*1/*β*2 integrin-mediated cell migration and adhesion [57]. Indeed, KF strongly suppressed the production and expression of inflammatory mediates such as NO, PGE_2_, and TNF-*α* in LPS-activated macrophages (Figures 1(b) and 2(a)). The adhesion event of U937 cells to FN was also dose-dependently diminished by KF (Figure 1(e)). Interestingly, the suppressive activities of these enzymes by KF, as measured by phosphorylation levels of the enzymes, were also found in stomachs treated with HCl/EtOH and pancreas exposed with LPS/CA (data not shown), implying that these enzymes play a central role in many different types of* in vivo* inflammatory symptoms, regardless of inflammatory stimuli. In fact, it is known that damage-associated molecular patterns including HMGB1 and ATP, which are released by sterile, damaged conditions, and pathogen-associated molecular patterns including LPS and peptidoglycan share TLR signaling pathways to generate cellular inflammatory responses [58, 59]. Thus, tissue and cellular damage that occurs under both infected and sterile conditions can induce the activation of NF-*κ*B and AP-1 via pattern recognition receptors such as TLRs in a similar manner [58]. Previous reports [60–62] and our data strongly indicate that the activation of Src, Syk, IRAK1, and IRAK4 is present in both* in vitro* and* in vivo* inflammatory models (Figures 3 and 4, data not shown). In addition, KF strongly inhibits the phosphorylation and subsequent enzyme activity of Src, Syk, IRAK1, and IRAK4, which is linked to its anti-inflammatory action (Figures 3 and 4). Several papers have speculated that KF might bind to the ATP binding sites of kinases, increasing ATP levels attenuated by the inhibitory potency of KF [54, 63], although identification of the amino acids of the ATP binding domain that are affected by KF remains unclear and needs further studies. This property could allow KF or other flavonoids broad-spectrum pharmacological activities in numerous molecular and cellular responses. It is known that steroid-backbone drugs (e.g., prednisolone or saponins) display variety of pharmacological actions by antagonizing intracellular glucocorticoid receptor [64, 65]. However, the fact that there is additive NO inhibitory activity during combination treatment of KF with prednisolone (Figure 1(d) right panel) seems to indicate that KF-mediated direct enzyme inhibition and steroid drug-mediated glucocorticoid receptor antagonism are distinctive pharmacological actions.

**Figure 5:**
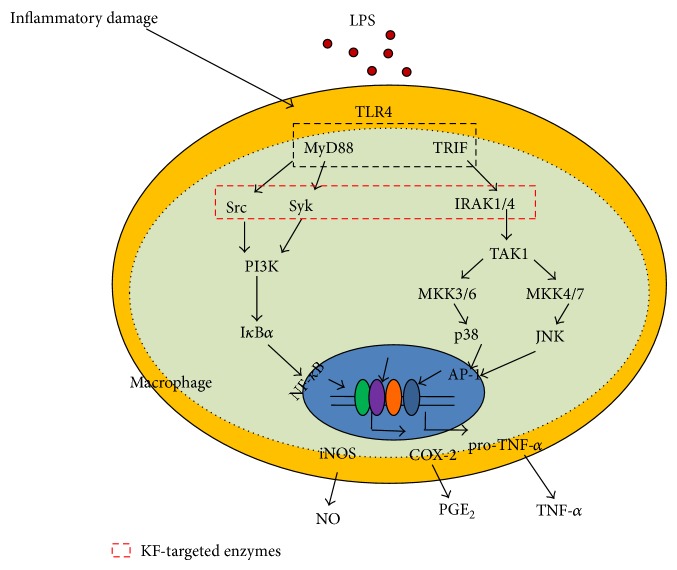
Putative KF inhibition pathway in macrophage-mediated inflammatory signaling events.

In summary, we have shown that KF is capable of effectively suppressing* in vitro* inflammatory responses as well as its radical scavenging activity. In particular, it was identified that KF serves as a direct inhibitor of Src, Syk, IRAK1, and IRAK4, playing a central role in the activation of NF-*κ*B and AP-1 as summarized in Figure 5. Since KF is included in many edible plants and fruits, we propose that KF-rich fractions from edible sources could be applied for the development of functional foods with anti-inflammatory properties.

## Figures and Tables

**Table 1:** Real-time PCR primers used in this experiment.

Name	Sequence (5′ to 3′)
Real-time PCR	
iNOS	
F	GGAGCCTTTAGACCTCAACAGA
R	TGAACGAGGAGGGTGGTG
TNF-*α*	
F	TGCCTATGTCTCAGCCTCTTC
R	GAGGCCATTTGGGAACTTCT
COX-2	
F	GGGAGTCTGGAACATTGTGAA
R	GCACATTGTAAGTAGGTGGACTGT
GAPDH	
F	CAATGAATACGGCTACAGCAAC
R	AGGGAGATGCTCAGTGTTGG

## Abbreviations

PG: Prostaglandin
NO: Nitric oxide
COX: Cyclooxygenase
iNOS: Inducible NO synthase
TNF-*α*: Tumor necrosis factor *α*
ERK: Extracellular signal-related kinase
TLR: Toll-like receptors
MAPK: Mitogen activated protein kinase
NF-*κ*B: Nuclear factor-*κ*B
AP-1: Activator protein-1
JNK: c-Jun N-terminal kinase
EIA: Enzyme immunoassay
ELISA: Enzyme linked immunosorbent assay
MTT: 3-(4,5-Dimethylthiazol-2-yl)-2,5-diphenyltetrazolium bromide (a tetrazole)
IRF-3: Interferon regulatory factor-3
DTT: Dithiothreitol
PI3K: Phosphoinositide 3-kinase
LPS: Lipopolysaccharide
RT-PCR: Reverse transcriptase-polymerase chain reaction.
